# Case Report: Persistent erectile dysfunction in a man with prolactinoma

**DOI:** 10.12688/f1000research.5743.1

**Published:** 2015-01-15

**Authors:** Justin Badal, Ranjith Ramasamy, Tariq Hakky, Aravind Chandrashekar, Larry Lipshultz

**Affiliations:** 1 Department of Urology, Baylor College of Medicine, Houston, TX 77030, USA

**Keywords:** prolactin, sexual dysfunction

## Abstract

Erectile dysfunction has been explored as a condition secondary to elevated prolactin; however, the mechanisms by which elevated prolactin levels cause erectile dysfunction have not yet been clearly established. We here present a patient with a history of prolactinoma who suffered from persistent erectile dysfunction despite testosterone supplementation and pharmacological and surgical treatment for the prolactinoma.  Patients who have had both prolactinemia and erectile dysfunction have been reported in the literature, but we find no report of a patient with persistent erectile dysfunction in the setting of testosterone supplementation and persistent hyperprolactinemia refractory to treatment. This case provides evidence supporting the idea that suppression of erectile function occurs in both the central and peripheral nervous systems independent of the hypothalamic-pituitary-gonadal axis.

## Introduction

Prolactinomas are the most common type of pituitary adenoma and account for 30% of all clinically recognized cases of pituitary adenomas ^1^. Generally, prolactinomas arise in the second to fourth decade of life and are more quickly recognized in women than in men because the women experience an abrupt cessation of menses ^1– 3^. In men, hyperprolactinemia (HPRL) causes hypogonadotropic hypogonadism leading to decreased libido, impotence, infertility, gynecomastia or galactorrhea ^1, 3, 4^. This effect is due to prolactin’s inhibitory action on gonadotropin releasing hormone, which ultimately results in decreased luteinizing hormone levels and decreased testosterone production by the testes ^1, 2, 4^.

## Case report

The patient is a 23-year-old male who was seen initially in our clinic because of bilateral nipple discharge. His past medical history included a prolactinoma initially diagnosed when, at the age of 15, he reported changes in his vision that were worst on the right side, with intermittent complete darkening. He was subsequently found to have a pituitary macroadenoma on imaging and a prolactin level of approximately 4000 ng/ml. After therapy with cabergoline 0.5mg, 3 times per week, his prolactin decreased to 30–40 ng/ml, but there was no increase in testosterone. He was given supplemental testosterone therapy with testosterone cypionate. Three years after initial diagnosis, his prolactin levels had increased to 1500 ng/ml, despite therapy with cabergoline.

He underwent transphenoidal resection 4 years after diagnosis (age 19) with removal of the sellar bulk of tumor, but with residual right cavernous tumor seen on post operative imaging. The prolactin levels decreased from 1500 to 300 ng/ml, but continued to rise over the next few months despite therapy with cabergoline. Because of worsening symptoms a year later (age 20), he had gamma knife therapy of the residual right cavernous portion. However, prolactin remained elevated in the 500 – 600 ng/ml range, even though he was receiving cabergoline.

When he came to our clinic, he could not achieve an erection sufficient for masturbation or sexual activity, and he did not have nocturnal erections. With adequate testosterone supplementation and anastrazole therapy, he experienced increased energy levels and elevated libido and has expressed the desire for treatment for his erectile dysfunction.

## Clinical findings

On physical examination, the patient was well developed and well nourished. His external chest exam was significant for gynecomastia despite recent bilateral mastectomy. The testes were 12cc in volume.

## Diagnostic assessment

Hormone testing revealed a prolactin level of 300 ng/ml (normal range is 3.0 – 30.0 ng/ml) while he was receiving therapy with cabergoline. Luteinizing and Follicle Stimulating Hormone remained low at all points during treatment ranging between 0.00 to 0.22 mIU/ml (normal range, 1.2 – 7.8 mIU/ml) and 0.09 to 0.36 mIU/ml (normal range, 1.3 to 11.4 mIU/ml), respectively. The patient’s testosterone ranged from 384 – 1600 ng/dl (normal range of 200 – 1000 ng/dl) while receiving testosterone supplementation with testosterone cypionate (200mg IM injection once a week initiated at the initial clinic visit).

## Therapeutic intervention

A duplex penile ultrasound following injection of 0.30cc of TriMix demonstrated some engorgement of the patient’s penis, with forty percent rigidity and no venous leak. Peak systolic velocity and end diastolic velocity were within normal limits. Despite treatment with tadalafil 5mg daily, he did not notice improvement in erectile function. He is now using a vacuum erection device for sexual activity. He was offered several different methods for improving his erectile function, including intracavernosal injections and an inflatable penile prosthesis and is currently debating which option to choose. Ultimately, the patient desires a more permanent solution to his erectile dysfunction, having stated that he is generally not satisfied with his current medical management for the erectile dysfunction. He notes that he is otherwise very pleased with the effects of the testosterone supplementation.

## Discussion

In 2013, a published case study described a man with a pituitary adenoma who reported loss of libido and inability to have an erection over a period of 8 years ^5^. All results of routine laboratory tests, including serum testosterone levels, were normal. Contrast MRI demonstrated a homogenous enhancement of the pituitary, and the patient was found to have a significantly elevated prolactin level. Despite normal testosterone levels, he had significant erectile dysfunction. After treatment with cabergoline, the patient achieved normal erectile function and improvement in neurologic symptoms. Similar to our patient, this patient had erectile dysfunction and hyperprolactinemia despite normal levels of testosterone, suggesting that prolactin has different ways of exerting its effect on erectile function.

**Figure 1.**
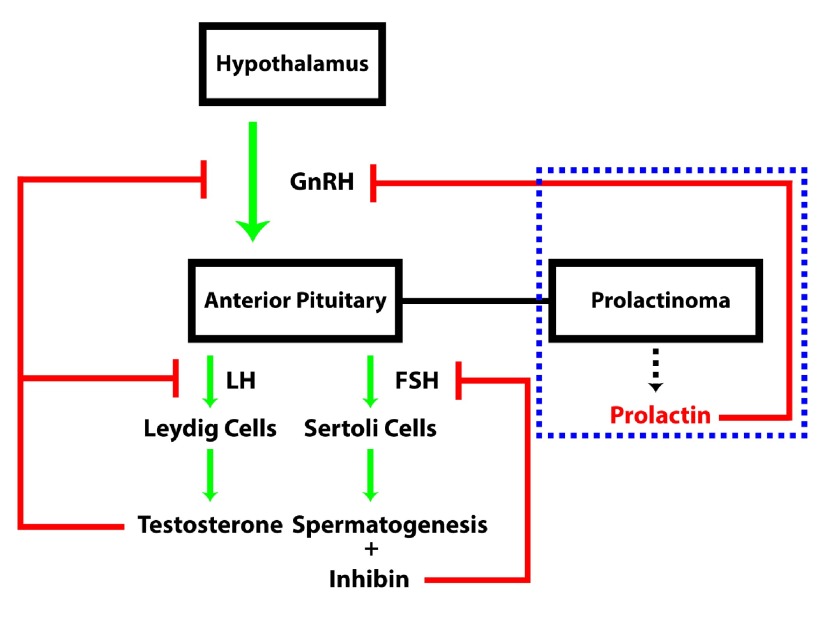
The normal hormonal axis regulating testosterone production. Gonadotropin releasing hormone (GnRH) stimulates release of Luteinizing Hormone (LH) and Follicle Stimulating Hormone (FSH) from the anterior pituitary which stimulates Leydig cells and Sertoli cells in the production of testosterone and sperm, respectively. Inhibin, produced by the Sertoli cells, negatively feeds back on FSH release from the anterior pituitary. Testosterone negatively feeds back on GnRH release from the hypothalamus and LH from the anterior pituitary, self-regulating its levels. In the dashed blue box, elevated prolactin secretion caused by a prolactinoma leads to pathologic inhibition of GnRH release from the hypothalamus and downstream inhibition of testosterone synthesis.

The relationship between hyperprolactinemia and testosterone was first described in 1978 when a group of hypogonadal men with hyperprolactinemia were reported to have regained their sexual function only after receiving bromocriptine therapy, even though they had previously received adequate amounts of supplemental testosterone ^6^ ( Figure 1). Another study in 2004 evaluated the effect of cabergoline on men with hyperprolactinemia and erectile dysfunction. The study measured episodes of nocturnal penile tumescence (NPT) and found that when hyperprolactinemic hypogonadic men were treated with cabergoline, the number of monitored erections during sleep (NPT) increased ^7^.

Unfortunately, there has been a misunderstanding of the hypothalamic-pituitary-gonadal axis with regard to erectile function and prolactin. Elevated levels of prolactin lead to an impairment of the pulsatile release of luteinizing hormone resulting in decreased serum testosterone secretion. This hypogonadism was generally accepted as the main cause of erectile dysfunction. However, as we observed, despite adequate supplementation of testosterone as initial therapy for erectile dysfunction, the patient continued to be unable to have an erection. This mixed picture of elevated prolactin and “normal” testosterone raises the question of where prolactin is exhibiting its inhibitory effect.

**Figure 2.**
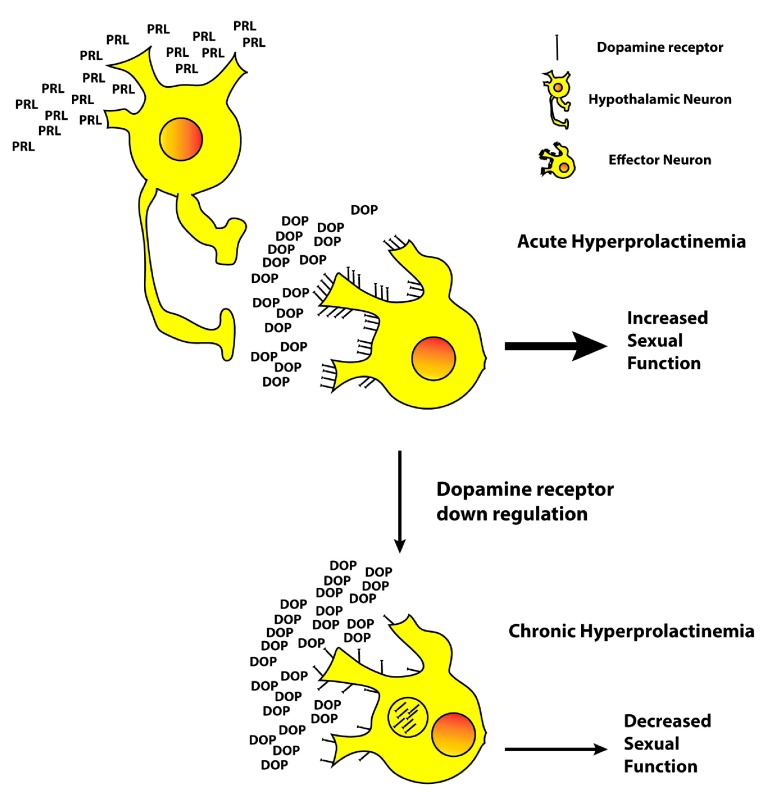
Schematic illustration of dopamine (DOP) receptor down regulation in a chronic hyperprolactinemia setting. Hypothalamic neurons are stimulated by chronically elevated levels of prolactin to excessively release dopamine which results in dopamine receptor internalization and dysregulation of the downstream signal for sexual function.

Prolactin exerts its effects in a variety of locations, including the hypothalamus and the cavernosal bodies ^8^. Hyperprolactinemia leads to increased expression of tyrosine hydroxylase mRNA in regions of the hypothalamus associated with sexual and erectile function. The fact that prolactin regulates the synthesis, release, and turnover of dopamine in hypothalamic neurons explains the initial increase in libido and erectile function observed in a study of male rats with acute hyperprolactinemia; however, a subsequent decrease in erectile function was observed as prolactin levels remained elevated, suggesting a down regulation of dopamine receptors secondary to chronically elevated levels of prolactin ( Figure 2). This potential dysregulation of dopamine could explain the central inhibitory effect of hyperprolactinemia on erectile function, especially since erectile function has been shown to be corrected following bromocriptine administration before hypogonadism has been adequately treated. This evidence points to prolactin exerting its effect outside of the hypothalamic-pituitary-gonadal axis and acting independently of depressed GnRH, LH, or testosterone levels.

Our patient had experienced a prolonged course of hyperprolactinemia and, with indicated treatment, did not adequately recover erectile function. Although the testosterone supplementation resulted in resolution of his other hypogonadal issues – namely loss of energy and mood swings – the elevated prolactin may have severely altered the excitatory pathways in the hypothalamus, necessitating longer term or more invasive treatment to ameliorate the offending suppression and erectile dysfunction.

## Consent

Written informed consent for publication of clinical details was obtained from the patient.
