# Supplementary material for: Case Report: Persistent erectile dysfunction in a man with prolactinoma
Source: F1000Res. 2015 Jan 15;4:13. [Version 1] doi: 10.12688/f1000research.5743.1 (PMC4367515; doi:10.12688/f1000research.5743.1)
Supplement: Peer review history

# Peer review: Referee response for version 1

**DOI:** 10.5256/f1000research.6139.r7928

**Published:** 2015-03-12

**Authors:** Ryan Smith

**Affiliations:** 1 Department of Urology, University of Virginia, Charlottesville, VA, USA

The authors have composed an excellent case report highlighting the fact that multiple mechanisms can impair erectile function. In addition, hormone replacement is not typically sufficient to resolve this issue. The pathophysiology behind the inhibitory effect of prolactin appears to extend beyond hypogonadism and is intriguing.

1. Do the authors have any IIEF/SHIM data at baseline and follow-up?

2. Perhaps the authors can comment on the use of TST as opposed to alternative agents (HCG) which would not impact fertility.

3. I would add some additional comments regarding the physical exam characteristics (i.e. secondary sex characteristics, etc.)

4. Do the authors have any conjecture as to the mechanisms underlying the impact of hyperprolactinemia on this patients ED despite TST?

I have read this submission. I believe that I have an appropriate level of expertise to confirm that it is of an acceptable scientific standard.

# Peer review: Referee response for version 1

**DOI:** 10.5256/f1000research.6139.r7689

**Published:** 2015-02-25

**Authors:** Wayland Hsiao

**Affiliations:** 1 Department of Urology, Emory University, Atlanta, GA, USA

This is an excellent case report that raises the idea that prolactin may have other mechanisms of action for suppression of erectile function besides the most commonly cited reason of low testosterone.

This case highlights the idea that there are certainly multiple mechanisms by which any biological alteration (high prolactin) can affect normal physiology (erectile function).

I would hope that the authors will comment on whether there are any plans to further intervene in this young man's prolactin beyond the already mentioned therapies. Also, for the evaluation of erectile function, more information could be given on the specific hemodynamic parameters obtained as well as any mention of what kind of baseline sexual function he has (either an IIEF score or other standardized questionaire).

However, overall, a great report to highlight that we don't understand all perturbation that go one when hormones are abnormal, and the "traditional" thinking of single pathways is probably folly and more a representation of reading about a disease in a textbook rather than critically thinking about the pathophysiology and the limitations of our current medical knowledge.

I have read this submission. I believe that I have an appropriate level of expertise to confirm that it is of an acceptable scientific standard.

# Peer review: Referee response for version 1

**DOI:** 10.5256/f1000research.6139.r7330

**Published:** 2015-01-29

**Authors:** Nelson Bennett

**Affiliations:** 1 Institute of Urology, Lahey Clinic Medical Center, Burlington, MA, USA

This is an interesting case report involving a young man with persistent erectile dysfunction in the setting of a prolactinoma.  

Introduction:

- What are some of the actions of prolactin in normal male physiology?

Case Report:

- The word "worst" is used inappropriately.
- Why was a 15 year old male started on testosterone cypionate instead of HCG or other medications that would not compromise his fertility?
- Are there plans for continued treatment of the prolactinoma? 

Clinical Findings:

- Please greatly expand this section.  What are his vital signs, visual acuity, body habitus, body hair distribution, tanner stage, penile size, etc?

Diagnostic Assesment:

- Aside from the prolactin, LH, FSH, and Testosterone levels, what is his TSH level and hematocrit level? 

Therapeutic Intervention:

- Was the duplex penile U/S completed in a multidosing regimen?
- What were the actual duplex parameters..i.e. the  PSV, EDV, and RI?

Discussion:

- No issues, however, the integrity of the manuscript would be enhanced if the authors would offer suggestions on how to assess and treat these complex patients.  

I have read this submission. I believe that I have an appropriate level of expertise to confirm that it is of an acceptable scientific standard, however I have significant reservations, as outlined above.
